# Transitioning to a digital TB case-based surveillance system: a mixed-methods study of the DHIS2 TB e-Tracker system

**DOI:** 10.5588/ijtldopen.25.0589

**Published:** 2026-03-13

**Authors:** M.L. Darboe, W. Samateh, S. Jaiteh, L. Badjie, L. Njie, B. Jassey, A. Bah, M. Njie, S. Jarjusey, C.K. Houessinon, C.S.C. Merle, V. Veronese

**Affiliations:** 1 Ministry of Health, the Gambia, West Africa;; 2 National HIV/AIDS Secretariat, the Gambia, West Africa;; 3 West & Central African Regional Network for TB, Cotonou, Benin;; 4 The Special Programme for Research and Training in Tropical Diseases (TDR), World Health Organization, Geneva, Switzerland.

**Keywords:** tuberculosis, Gambia, District Health Information System 2, TB surveillance

## Abstract

**BACKGROUND:**

TB surveillance remains critical to disease control. The Gambia transitioned to an electronic, case-based surveillance from monthly aggregate reporting. This study assessed the knowledge, attitudes, practices, data accuracy, concordance, and system usability following the e-Tracker pilot.

**METHODS:**

A mixed-methods study was conducted across 11 sites over 6 months. Quantitative data were collected from the District Health Information System 2 aggregate and e-Tracker to calculate verification factor and concordance of data, and in-depth interviews were conducted to understand perceptions, usability challenges, and implementation barriers. Data were analysed using descriptive statistics and thematic analysis.

**RESULTS:**

Over two thirds of users demonstrated understanding of case-based surveillance. Overall concordance between reports and e-Tracker at the national level was high, and lower accuracy was observed at the facility. While the e-Tracker enabled richer data capture for key indicators, routine use for patient management was limited. Key barriers included inadequate infrastructure, staff turnover, dependence on single users, and variable digital literacy. Most participants reported improved efficiency and optimism.

**CONCLUSION:**

The e-Tracker shows promise for strengthening TB surveillance. To realise its impact, scale-up must capture infrastructure, capacity building, and inputs from frontline staff to ensure sustainability. The study is limited by reliance on self-reported experiences and absence of long-term outcome data.

The transition from paper-based, aggregate reporting to electronic case-based surveillance is essential for improving the efficiency and effectiveness of TB control efforts,^1^ and to enable timely and accurate data to inform public health decisions.^2^ Advantages of case-based surveillance include enhanced data quality, easier and more timely access to information, and the generation of individual-level data for better understanding TB transmission dynamics and enhanced patient management and follow-up.^3-5^ In The Gambia, TB data are reported monthly by health facilities as aggregated data, then entered into the District Health Information System 2 (DHIS2), which takes time and limits access and availability for effective national-level decision-making and response strategies. To address these challenges, the National Leprosy & Tuberculosis Control Program and partners implemented the e-Tracker, a digital case-based surveillance system, which was piloted at various facilities to improve the quality, timeliness, and utility of TB data.^6^ This study evaluated the pilot implementation by assessing the knowledge, attitude, practice, data accuracy, and concordance of e-Tracker against the aggregate to inform the larger rollout in the Gambia.

## METHODS

A mixed-methods design comprising prospective TB surveillance data collection from TB service points (DOTs) and qualitative interviews to assess e-Tracker users’ knowledge, attitudes, and practices (KAP) was conducted in 11 DOT sites from 1 June to 30 November 2024.

### Setting

The Gambia, located in West Africa, is the smallest country on the mainland with an estimated population of 2.9 million.^7^ The prevalence of TB is 142 per 100,000 population.^8^ TB services are delivered within the Primary Health Care system, through a network of health facilities across the country. For this study, TB sites were selected based on the 2022 case notification data. Sites that together accounted for about 80% of the national TB notifications were included, ensuring representation of both aggregated and case-based reporting. At least one DOT centre was selected from each of the seven regions, with emphasis on facilities managing higher caseloads.

### Intervention

E-Tracker is an electronic TB case-based tracker, developed by the Ministry of Health and adapted from the WHO DHIS2 case-based TB package.^9^ The tracker records individual-level data on all notified TB cases across participating sites and supports the use of information at facility, regional, and national levels for patient management and programme decision-making, and the system allows offline data entry with automatic synchronisation when connectivity is available. Key features include automatic reporting and patient tracking using multiple identifiers that enable staff and supervisors to monitor service delivery and patient follow-up. From June to November 2024, 11 TB treatment sites were transitioned to a hybrid reporting system, where cases were entered daily into the DHIS2 TB tracker, while paper registers continued to generate monthly reports for submission into the DHIS2 aggregate system. The e-Tracker captured socio-demographic characteristics, type of TB, site of disease, and TB/HIV indicators (testing, enrolment on anti-retroviral therapy [ART], and cotrimoxazole preventive therapy). The system has the capacity to record risk factors such as smoking, alcohol use, and nutritional status, and these were not used for comparison.

### Study outcomes

The primary outcomes were the KAP of e-Tracker users, as well as the accuracy and concordance of data captured by the e-Tracker compared to the aggregate system. KAP was explored qualitatively through interviews with e-Tracker users to contextualise the quantitative findings. Data accuracy and concordance were assessed quantitatively by comparing the e-Tracker with aggregate reports. Accuracy was defined as the absolute difference between verified and reported values, expressed as a verification factor (VF), calculated as the ratio of verified (benchmark) to reported data. VF is expressed as a percentage for patient-level variables to reflect proportional differences, and as raw differences for facility-level case counts to highlight absolute discrepancies relevant for programmatic evaluation. Concordance levels were graded as follows: 0% difference (perfect match), ±5% (acceptable), ±10% (minor variation), and >±10% (major variation).

### Participants

Eligible participants for the KAP component were individuals directly involved in TB service delivery and management at the facility, as well as the regional TB supervisor. Purposive sampling was applied to capture a range of perspectives across different facility levels and geographic regions. Participants were recruited from all 11 TB sites.

For the assessment of accuracy and concordance, the line list of all patients in the facility TB register and the patient record cards were used from June to November 2024 for data extraction.

### Procedures

Data entry into the e-Tracker using pre-installed DHIS2 e-Tracker tablet and the use of customised data collection tools for the aggregated data were performed at the facility level by designated TB focal persons, trained during the pilot. Each Regional Leprosy/TB Control Officer supervised data entry quality and provided ongoing support. The following indicators were identified for comparative analysis: notified TB cases, age of respondents, type of TB based on site and treatment history and TB/HIV testing, positivity, and ART enrolment. The qualitative interviews were conducted at the facility by a trained Field Epidemiologist and an M&E officer outside the Ministry of Health. Participants were interviewed in September 2024 (after 3 months of using e-Tracker).

### Data collection, management, and analysis

Data collectors used tablets pre-installed with the DHIS2 tracker, designed explicitly for this study. Upon patient enrolment, information from the facility TB register was uploaded to the e-Tracker. Regional Leprosy/TB Control Officers supervised data entry at their assigned facilities through customised dashboards. Data were extracted using TB programme indicators configured in the e-Tracker and aggregate system. Quantitative analysis was conducted using Microsoft Excel and STATA® (v17.0), applying basic descriptive statistics to calculate study outcomes. Qualitative interviews were transcribed, and a grounded theory approach was used to identify emergent themes, which coders applied to the transcripts using ATLAS.ti.

### Ethical statement

This study received approval from the Joint Gambia Government and MRC Research Ethics Committee (Ref: 30134).

## RESULTS

Data from 682 patients across 11 facilities were collected and compared between the validated aggregated data and the e-Tracker. Overall, most patients were male (66%), aged between 15 and 34 years (23%), new TB patients (93%), pulmonary, and HIV negative. Although variance was found between key variables across the two sources, the most notable variance was child TB notification, with a VF of 61% (over-reporting in e-Tracker) for very young children and 173% (under-reporting in the e-Tracker) for older children. Additionally, a higher proportion of missing data was noted in the e-Tracker compared to the reference dataset, especially for treatment history and HIV status. Variables such as sex and site of TB disease were similar across both systems (Table 1).

**Table 1.** Socio-demographic and risk factor characteristic of TB patients (DHIS2 aggregate versus DHIS2 e-Tracker.

Variable	DHIS2 aggregate	DHIS2 e-Tracker	VF
Sex of enrolled patients	n (%)	n (%)	
Male	449 (66%)	467 (67%)	96%
Female	233 (34%)	228 (33%)	102%
Missing	0	2	
Age of enrolled patients			
Mean (SD)	N/A	36 (±17)	N/A
0–4	17 (2%)	28 (4%)	61%
5–14	26 (4%)	15 (2%)	173%
15–24	154 (23%)	145 (22%)	106%
25–34	164 (24%)	169 (24%)	97%
35–44	119 (18%)	125 (18%)	96%
45–54	92 (13%)	92 (13%)	100%
55–64	66 (10%)	75 (11%)	88%
≥65	44 (6%)	45 (6%)	98%
Missing	0	3	
Type of TB (registration group)			
New	633 (93%)	582 (84%)	109%
Relapse	32 (5%)	30 (4%)	107%
Previously treated	15 (2%)	15 (2%)	100%
Previously treated/unknown/missing	2	70 (10%)	3%
Type of TB (site of disease)			
Pulmonary	632 (93%)	632 (91%)	100%
Extra-pulmonary	50 (7%)	49 (7%)	102%
Missing	0	16 (2%)	
Tested for HIV before or during TB treatment			
Status known	674 (99%)	662 (95%)	102%
Unknown	8 (1%)	13 (2%)	62%
Missing	0	22 (3%)	
HIV status of TB patients			
Positive	95 (14%)	101 (15%)	94%
Negative	579 (86%)	561 (85%)	103%
HIV-positive TB patients on ART			
Yes	84 (88%)	57 (65%)	147%
No	11 (12%)	16 (16%)	69%
Missing	0	28 (28%)	

DHIS2 = District Health Information System 2; VF = verification factor; ART = anti-retroviral therapy.

A comparison of DHIS2 aggregate data and DHIS2 e-Tracker records across 11 health facilities revealed overall acceptable concordance, although variability was observed between sites. Two facilities (18%), both classified as rural and medium TB burden, showed complete concordance between the two systems. Six facilities (55%), including two with high TB burden, demonstrated acceptable levels of concordance. Minor discrepancies were observed in two urban facilities, while one rural facility exhibited a significant discrepancy between the systems. Notably, the facility with the significant discrepancy employed staff with the highest average years of experience (26 years) and age (50 years) among all sites (Table 2).

**Table 2.** Facility-level comparison of TB case notifications from DHIS2 aggregate and e-Tracker with concordance grading.

Facility	Location	Staffing number	Average years of experience	TB burden	DHIS2 aggregate	DHIS2 e-Tracker	Difference	% difference	Grading
Bansang Hospital	Rural	2	25	Low	24	51	−27	−113%	Major. variation
Basse District Hospital	Rural	2	18	Medium	38	38	0	0%	Perfect match
Brikama District Hospital	Urban	4	13	High	188	195	−7	−4%	Acceptable
Bundung Maternal Child Health Hospital	Urban	2	23	Medium	76	72	4	5%	Acceptable
Bwiam General Hospital	Semi-urban	2	10	Low	22	23	−1	−5%	Acceptable
Essau District Hospital	Rural	2	16	Medium	28	29	−1	−4%	Acceptable
Farafenni General Hospital	Rural	5	21	Medium	41	39	2	5%	Acceptable
Kanifing General Hospital	Urban	4	12	High	69	66	3	4%	Acceptable
Sanyang Community Clinic	Urban	2	11	Low	14	13	1	7%	Minor variation
Soma District Hospital	Rural	3	24	Medium	26	26	0	0%	Perfect match
Sukuta Min. Health Centre	Urban	2	13	High	156	145	11	7%	Minor variation
Total					682	697	−15	−2%	Acceptable

DHIS2 = District Health Information System 2.

Table 3 presents the socio-demographic characteristics of the respondents who participated in the qualitative interviews. The mean age was 41 ± 10 years. Most respondents were male (10, 77%), with the majority serving as Leprosy/TB Inspectors (62%) and classified as nurses by professional cadre (85%). On average, participants had 18 ± 11 years of professional experience.

**Table 3.** Socio-demographic characteristics of study respondents (qualitative).

Variable	n (SD) (%)
Age of respondents	
Mean	41 (±10)
Min	26
Max	59
Sex of respondents	
Male	10 (77%)
Female	3 (23%)
Cadre	
LTI	8 (62%)
RLTCO	5 (38%)
Profession	
Nurse	11 (85%)
Social worker	2 (15%)
Years of experience	
Mean	18 (±11)
Min	3
Max	30

LTI = Leprosy TB Inspector; RLTCO = Regional Leprosy and Tuberculosis Control Officer.

### Understanding of TB case-based surveillance

Over two thirds of respondents understood TB case-based surveillance as recording patient information in a digital system. One participant explained,

My understanding of TB case surveillance is that when you have a case and record that patient in a device or a system, that constitutes TB case-based surveillance. We make sure they are put on the right treatment.

This view focused mainly on tracking patient numbers and clinical variables. In practice, however, respondents noted that the e-Tracker was primarily used for central monitoring rather than for patient management at facilities. As a respondent from Bansang put it,

My understanding of TB surveillance is that they want to know how TB is going and how many cases there are present, whether TB is reducing or increasing. This involves entering the TB tracker, utilising the TB record-keeping tools for monitoring by the teams.

### Perceived benefits

Almost all respondents described the TB e-Tracker as a valuable tool with potential benefits such as real-time data sharing, improved clinical management, and enhanced programme oversight. Many emphasised its role in helping supervisors track service delivery and respond quickly to changes in case numbers. As one participant from the Lower River Region noted,

It is very good because it will help both the supervisor and LTIs to monitor their data and know the progress of the patient.

Another added, ‘It is a way of giving information to our supervisor by logging into the system and getting all the information you need at your desk’.

### Perceived challenges

Nearly all respondents reported challenges in using the TB e-Tracker, including limited staff capacity, inadequate devices, poor internet connectivity, and frequent power outages. While at least one staff member per facility was trained, their absence often stalled enrolment, and even trained staff struggled with the system, leading to errors such as duplicate entries. As a health worker from Sukuta Health Center explained,

Some of the staff are not trained. Quality internet is a challenge. Increasing our salaries, providing materials like laptops, and the space (we have a small space) is required. Sometimes I struggle to view certain variables.

Another from the Central River Region added, ‘Internet is a problem for the device because you cannot use a laptop everywhere’.

### Thoughts/feelings on new system

Most respondents expressed optimism about the TB e-Tracker, linking their enthusiasm to the growing role of digital tools in health care. A participant from Kanifing General Hospital noted,

I'm very excited because technology is advancing.

Over two thirds highlighted its potential to improve service delivery by reducing time spent on data entry and minimising errors compared to paper-based methods, as one health worker from Western-II Region explained,

…the TB tracker will improve the service delivery by reducing the time spent on data entry. It can reduce errors.

However, some stressed that adoption requires internal knowledge sharing. A participant from Brikama District Hospital observed,

The fact that we are using a new system requires time to adapt. Some of my colleagues keep saying I am the only trained person and I have to do it.

This underscores the need for peer learning and wider staff engagement to maximise the system’s benefits.

### Reflections on transition

Many respondents expressed support for the TB e-Tracker, especially when key enablers were in place. Others were more cautious, citing incomplete implementation across facilities, which complicated patient referrals. A respondent from Sukuta Health Centre explained,

I'm not very excited since not all health facilities are implementing it. The staff has problems with the internet, and as a supervisor, I'm also facing challenges with data. They need to have a desktop or laptop.

This highlights the need for equitable rollout and infrastructure support to ensure smooth adoption and continuity of care.

## DISCUSSION

The findings of this study show that slightly more than two thirds of participants demonstrated an understanding of the TB e-Tracker. This was supported by a high level of concordance between verified data and entries in the e-Tracker, with a minimal discrepancy of only −2%, which falls within the acceptable range for routine data accuracy.^10^ However, qualitative insights revealed that the e-Tracker was rarely used for day-to-day patient management. Staff consistently described it as ‘mainly for reporting to the program’, emphasising its role in central oversight rather than supporting clinical decision-making at the facility level. This perception was reflected in the data, as only two facilities achieved a perfect match between verified records and e-Tracker entries, while facilities like Bansang Hospital showed major deviations, likely due to a combination of infrastructure challenges, including intermittent electricity and internet, and relatively older staff who faced difficulties navigating the e-Tracker. The large variance observed in child TB notifications may partly reflect differences in age determination, as the e-Tracker calculates age from exact dates of birth, whereas the aggregate system often relies on caregiver-reported estimates. If the e-Tracker had been routinely used to guide patient management, closer alignment between the two data sources would have been expected. Similar challenges have been observed in other contexts, where digital health interventions often prioritise data collection and programme reporting but offer limited utility for real-time clinical support, leading to low adoption for patient management.^11,12^ Nutley et al^13^ further argue that such patterns reflect a broader structural feature of health information systems in low- and middle-income countries, where data flows are primarily vertical and designed for upward accountability, with limited use of data for decision-making at the facility.

On usability and sustainability, duplicate entries were observed in more than 80% of the pilot facilities; infrastructure emerged as a fundamental barrier in the qualitative assessment. During the pilot, the e-Tracker tracked potential duplicates using multiple variables, but these rules were case sensitive and inconsistently applied across facilities. The inconsistent use of validation rules, inadequate access to functioning electronic devices, and unstable internet connectivity, among others, are commonly cited challenges that lead to multiple records of a single client, and this was consistent with earlier research on digital health system implementation in resource-constrained settings.^14,15^ These challenges create frustration among users, especially those with limited access to expertise to fix the encountered challenge. Considerations should be made for the use of the health insurance numbers once the scheme has been effected across facilities.

The findings also highlight the human resource dimension to system uptake. Although at least one staff member was trained in each facility during the pilot phase, their absence often led to the complete cessation of e-Tracker-related activities. This indicates both a lack of peer–peer mentorship and overreliance on a single point of expertise. Furthermore, even among trained staff, difficulties understanding system variables and navigation led to data quality issues such as duplicate entries. Recommendations from previous experiences indicate that more sustainable capacity building initiatives should be explored, such as peer-to-peer learning, mentorship, and supportive supervision, especially at the regional level.^16^

Despite these challenges, many respondents expressed optimism about the potential of the system. Over two thirds of participants noted that the e-Tracker could improve service delivery by reducing the time spent on manual data entry and minimising errors commonly associated with paper-based systems. They also highlighted its potential to facilitate real-time data sharing, improve continuity of care, and support timely decision-making by supervisors.^17^ These findings align with global evidence on the benefits of digital health tools in improving data accuracy, accelerating information flows, and enabling more responsive health systems. However, the realisation of these benefits hinges on the presence of key enablers, including adequate equipment, robust connectivity, competent human resources, and ongoing managerial support.^18^

Variability in prior exposure to similar systems was evident, suggesting differing levels of digital readiness. For some, the tracker was entirely new, and for others, it represented an evolution of the paper-based tool. This underscores the need for context-sensitive implementation strategies that account for the existing capacity, digital literacy, and workload of users.^19^ It is essential to position digital health tools like the TB e-Tracker as clinical assets and not just administrative systems by embedding them into the routine service delivery workflow and ensuring frontline workers generate some value from the system. Investment in the infrastructure needed for optimal functioning of the TB e-Tracker should be prioritised to ensure uninterrupted use. Capacity building initiatives should be ongoing, layered, and supported by mentorship and feedback loops to ensure sustained capacity. Finally, engaging the health care workers in system design and refinement can help ensure that the tool aligns with their needs.

While this study provides in-depth insights into user experiences with the TB e-Tracker, it is limited by its reliance on self-reported perceptions, which may be subject to social desirability or recall bias. Further studies can explore the long-term benefit of the system by assessing improvements in patient outcomes.
